# Tailoring CsPbBr_3_ Growth *via* Non-Polar Solvent Choice and Heating Methods

**DOI:** 10.1021/acs.langmuir.2c01214

**Published:** 2022-07-21

**Authors:** Hediyeh Zamani, Tsung-Hsing Chiang, Kaylie R. Klotz, Annie J. Hsu, Mathew M. Maye

**Affiliations:** Department of Chemistry, Syracuse University, 111 College Place, Syracuse, New York 13244, United States

## Abstract

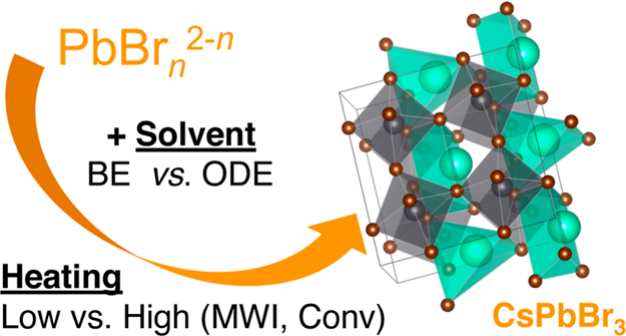

This study describes an investigation of the role of non-polar solvents on the growth of cesium lead halide (CsPbX_3_ X = Br and I) nanoplatelets. We employed two solvents, benzyl ether (BE) and 1-octadecene (ODE), as well as two nucleation and growth mechanisms, one-pot, facilitated by microwave irradiation (MWI)-based heating, and hot-injection, using convection. Using BE and MWI, large mesoscale CsPbBr_3_ nanoplatelets were produced, whereas use of ODE produced small crystallites. Differences between the products were observed by optical spectroscopies, which showed first band edge absorptions consistent with thicknesses of ∼9 nm [∼15 monolayer (ML)] for the BE-CsPbBr_3_ and ∼5 nm (∼9 ML) for ODE-CsPbBr_3_. Both products had orthorhombic crystal structures, with the BE-CsPbBr_3_ revealing significant preferred orientation diffraction signals consistent with the asymmetric and two-dimensional platelet morphology. The differences in the final morphology were also observed for products formed *via* hot injection, with BE-CsPbBr_3_ showing thinner square platelets with thicknesses of ∼2 ML and ODE-CsPbBr_3_ showing similar morphologies and small crystallite sizes. To understand the role solvent plays in crystal growth, we studied lead plumbate precursor (PbBr_*n*_^2–*n*^) formation in both solvents, as well as solvent plus ligand solutions. The findings suggest that BE dissolves PbBr_2_ salts to a higher degree than ODE, and that this BE to precursor affinity persists during growth.

## Introduction

All inorganic cesium lead halides (CsPbX_3_, X = Cl, Br, and I) and hybrid methylammonium lead halides (MAPbX_3_) are important functional materials^1−5^ that can be synthesized as crystals, thin films, and nanomaterials with varied morphologies.^6−14^ Two dimensional (2D) plates and platelets are common, with thicknesses of only a few monolayers (MLs) often observed, and lengths varied from nanometers to microns, which are formed during nucleation and growth, or self-assembled *via* solvent and or ligand-mediated interactions.^4,15−21^ Studies have revealed that control over ligand types and stoichiometry,^20^ as well as time and temperature of the synthesis can render various morphologies.^3,4,16,17,20,22−31^ Sonication,^32^ solvothermal,^17,33,34^ mechanochemical,^35^ and microwave irradiation (MWI)^4,36^ have also been employed to control CsPbBr_3_ growth. Studies have shown that Ostwald ripening^37,38^ and long-ranged growth that includes oriented attachment of smaller building blocks (cubes, rods, *etc.*) render 2D growth into nanoplatelets.^25,33,34,39^

The role that solvent plays in CsPbBr_3_ growth has been explored,^21,33,40−43^ and the role it plays in the dissolution and solvation of PbX_2_ salt into PbX_*n*_^2–*n*^ plumbate complexes. This is particularly interesting as the varied lead centered polyhedra formed will have different charges, molecular weights, solubility, as well as concentration and reactivity. The dynamic equilibrium between plumbates is sensitive to the Lewis basicity of the solvents and ligands involved, as well as temperature. Till date, studies have focused primarily on polar solvents and their role in plumbate formation, with nitrogen or sulfur bearing solvents (*e.g.*, DMF and DMSO) acting as stabilizers of PbX_*n*_^2–*n*^.^44^

In this report, we study whether non-polar, high boiling point solvents can tune CsPbBr_3_ growth at both low and high temperatures, with the latter being used to compare a one-pot growth mechanism, facilitated by MWI,^45,46^ and a hot-injection mechanism, *via* convection. The findings indicate that these solvents do tune PbX_*n*_^2–*n*^ formation, leading to controlled CsPbBr_3_ growth and properties.

## Experimental Section

### Chemicals

Lead iodide (PbI_2_, 99%), lead bromide (PbBr_2_, 99.99%), cesium carbonate (Cs_2_CO_3_, 97%), methyl ammonium iodide (MAI, 98%), oleyl amine (OAm, 70%, technical grade), oleic acid (OAc, 90%), 1-octadecene (ODE, 90%), benzyl ether (BE, 98%), and *N*,*N*-dimethylformamide (DMF, anhydrous, 99.8%) were purchased from Sigma-Aldrich and used as received unless otherwise noted.

### Precursor Preparation

In a typical one-pot reaction, precursors were first prepared by adding 0.80 g of Cs_2_CO_3_ to 30 mL of solvent (ODE or BE), along with 2.4 mL of OAc, and the mixture was heated at 120 °C under vacuum until all was dissolved (0.15 M Cs-OAc). Likewise, 0.28 g of PbBr_2_ or 0.34 g of PbI_2_ in 20 mL of solvent was heated at 120 °C for 1 h under vacuum. Next, 2 mL of OAc and 2 mL of OAm were added to the mixture under Ar, and the mixture was heated at 120 °C for another hour for a complete dispersion of a 0.03 M PbX_2_ stock solution. Trials were also run using purified OAm,^47^ as were reactions where Cs_2_CO_3_ was reacted with OAc at higher ratios.^48^

### One-Pot MWI Heating and Synthesis of CsPbX_3_

In a typical one-pot MWI heated reaction, 3 mL of the as-prepared PbX_2_ precursor was purged with Ar gas for 5 min at room temperature. Next, 200 μL of the cesium oleate precursor solution was heated to 85 °C and injected to PbX_2_ solution followed immediately by MWI heating to 160 °C where the trajectory of the heat transfer was different between BE and ODE and depends on the dielectric constant (ε) of the non-polar solvents.^49^ The BE reaction (ε = 3.82) reached 160 °C within 100 s, while the ODE reaction (ε = 2.25) reached after 240 s. Upon reaching the set point, there was a rapid quenching of temperature by the active cooling of the MWI reactor. These prepared nanoplatelets were purified by centrifuging 1 mL of aliquots at 10,000 rpm for 3 min, followed by the discarding of the supernatant and redispersion in toluene aided by sonication. This procedure was repeated at least two times, except in the case of Fourier transform infrared spectroscopy (FTIR) sample preparation, in which additional purification steps were used.

### Hot-Injection Synthesis of CsPbX_3_

First, a 0.15 M Cs-OAc precursor was prepared, as described in the precursor preparation section, and was stored at 85 °C prior to the injection. To prepare the PbBr_2_ precursor, a mixture of 0.14 g of PbBr_2_ powder and 10 mL of BE was heated under vacuum at 120 °C for 1 h. Then, the mixture was placed under Ar, and 1 mL OAm and 1 mL OAc were injected to dissolve the powder. After 1 h, temperature was raised to 160 °C. Next, 0.8 mL of Cs-OAc was injected to the solution, and the reactions were let to anneal for 1, 30, and 60 min before removing the heating mantle to quench the reaction. The products were purified, as described above. This method was also used for the room-temperature syntheses described, except that all reactants were cooled to room temperature before initiation.

### BE-CsPbI3 Synthesis *via* Halide Exchange of BE-CsPbBr_3_

Prior to the halide exchange (HE), BE-CsPbBr_3_ were purified and redispersed in toluene to approximated concentrations.^50^ This solution was then combined with an aliquot from a 0.20 M PbI_2_ stock solution so that the combined [I^–^]:[Br^–^] = 1, following a method recently developed in our laboratory.^51^ The mixture was allowed to react for 30 min, then photoluminescence (PL) emission was measured. For X-ray diffraction (XRD) measurement, a portion of the mixture was separated, purified, and drop-cast on a zero-diffraction substrate. The rest of the mixture was centrifuged and redispersed in toluene to the initial volume to remove the free Br^–^ in the solution. In the second step, PbI_2_ of the same ratio was added to the remaining solution, followed by XRD and PL measurements.

### Benesi-Hildebrand Analysis

The competitive assay analysis was performed by first preparing a 0.1 M MAI in BE. Next, MAI aliquots were added to a diluted BE-PbI_2_ at ratios of [MAI]/[PbI_2_] = 1-35. The resulting iodoplumbate complex formation was monitored by UV–vis spectroscopy.

## Instrumentation

MWI heating was performed using a Discover-SP microwave synthesizer (CEM Inc.) with a magnetron frequency of 2450 MHz, where temperature, power, and time were controlled by Synergy software. Each reaction was stirred in a 10 mL glass vial during which temperature was monitored by a IR sensor. To stop the reaction, samples were cooled by compressed N_2_ circulating inside the microwave chamber. The optical characterization was performed on a Cary 50 Bio UV–vis spectrophotometer (Varian Inc.), and PL spectroscopy was performed on a Cary Eclipse fluorescence spectrophotometer (Varian Inc.). The excitation wavelength was 400 nm. The powder XRD was performed using D2 PHASER XRD (Bruker Inc.) with a Cu radiation source. Samples were prepared by drop-casting purified products on a zero-diffraction quartz holder or by addition of dried powders. Transmission electron microscopy (TEM) was performed on either a JEM 2100F or JEM 1400 (JEOL Inc.), operated at 200 or 120 kV, respectively. Samples were drop cast from toluene dispersions onto carbon-coated copper grids. Atomic force microscopy (AFM) analysis was performed on an Innova SPM (Bruker Inc.) in the tapping mode using samples deposited on a freshly cleaved highly ordered pyrolytic graphite (HOPG) grid. Finally, FTIR spectra were collected using a Thermo Nicolet 6700 FTIR equipped with a diamond smart iTR attenuated internal reflectance accessory and a liquid N_2_ cooled MCT-A detector. Samples were prepared by three rounds of purification in toluene with centrifugation at 4 k rpm for 10 min in a glass tube. Samples were then drop-cast on the iTR diamond and air-dried prior to the measurement.

## Results and Discussion

**Figure 1:**
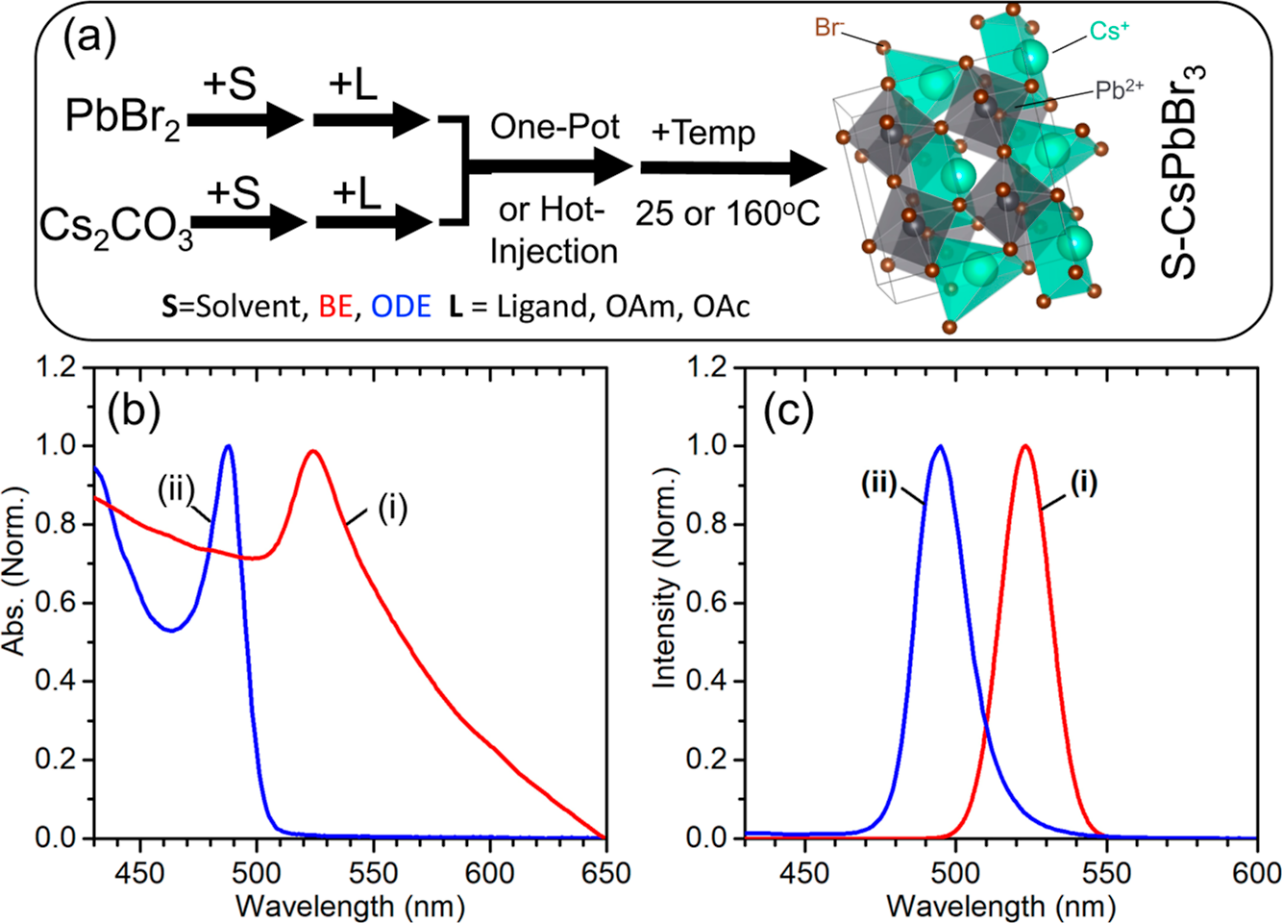
(a) Schematic overview of the reaction systems used in this study. UV–vis (b) and PL emission (c) spectra of the BE (i), and ODE-CsPbBr_3_ (ii) products prepared *via* MWI heating to 160 °C.

Figure 1a shows a schematic illustration of the synthetic conditions tailored to understand CsPbBr_3_ growth using either BE or ODE as non-polar high boiling point solvents, cesium carbonate dissolved and complexed with oleic acid (OAc) and lead plumbate (PbBr_*n*_^2–*n*^)^43,52−54^ formed *via* dissolving in solvent (S) and ligands (L), as described in the Experimental Section. The products of these reactions are denoted as BE-CsPbBr_3_ or ODE-CsPbBr_3_. Two nucleation and growth mechanisms were studied, one-pot and hot-injection, at either high temperature or low temperature.

A one-pot mechanism using MWI as the heating source was first employed, and the synthesis mixtures were heated to 160 °C. Upon cooling, the reaction solution had changed color from a light translucent yellow to a turbid orange, which if left overnight, would settle for the BE-CsPbBr_3_ products but not for the ODE-CsPbBr_3_. After collection and purification, the resulting optoelectronic properties were measured. Figure 1b shows the UV–visible absorption (UV–vis) results. The BE-CsPbBr_3_ had a first excitonic absorption peak at 523 nm (i), while the ODE-CsPbBr_3_ absorbed at 488 nm. Both products exhibited PL, as shown in Figure 1c, with BE-CsPbBr_3_ emitting at 527 nm (i) and ODE-CsPbBr_3_ at 495 nm (ii). The red-shift indicates either larger sizes or thicker platelets for BE-CsPbBr_3_. The band edge absorption is quantized by the minimum CsPbBr_3_ dimension, especially when sizes are comparable to the exciton Bohr radius, which for CsPbBr_3_ is ∼7 nm. Using the studies reported by others,^19,23,25,55^ we used this absorption to estimate a thickness of >15 ML, where ML is defined as a linear chain of corner sharing PbBr_6_^4–^ octahedra with thickness of 0.59 nm, for the BE-CsPbBr_3_, corresponding to approximate thickness of 8–9 nm. We note that the absorption is broad, indicating a polydisperse sample and distribution in thicknesses. Using the same approach, the ODE-CsPbBr_3_ would have a minimum feature size of ∼9 ML or ∼5.3 nm.

**Figure 2:**
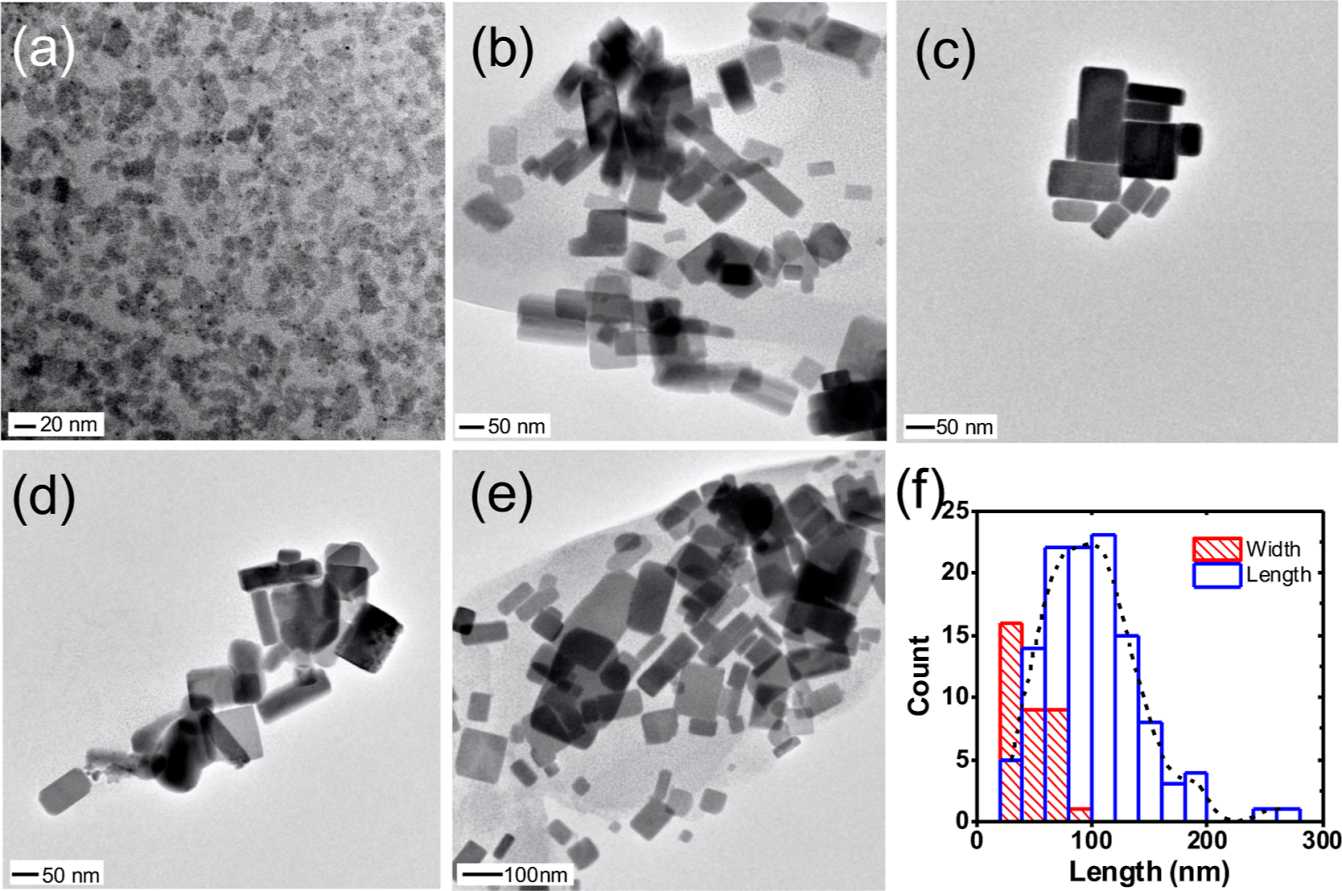
Representative TEM micrographs of ODE-CsPbBr_3_ (a) and BE-CsPbBr_3_ products (b-e). Size analysis histogram of BE-CsPbBr_3_ (f).

The dimensions and morphology of the two products were characterized using TEM. Figure 2a shows a micrograph for the ODE-CsPbBr_3_ products, which have small square-like lateral morphology with a length of ∼ 9.7 ± 0.8 nm, which combined with the optical data above is ∼5 nm thick and is consistent with other ODE-based CsPbBr_3_ platelets. In contrast, the BE-CsPbBr_3_ products had larger 2D platelet-like morphology, as shown in Figure 2b–e and Supporting Figure S1. The large platelet morphology of the products was highly reproducible; however, the lateral dimensions were polydisperse, with edge length variations of 20–500 nm (Figure 2f). The platelets were indeed thin, as indicated by the UV–vis, and observed in the TEM micrographs where the platelets orient on top of another.

The thickness was also probed by AFM. Figure S2 shows a typical AFM image of BE-CsPbBr_3_ platelets dropcast from a toluene dispersion onto a HOPG grid. At the resolution shown, domains consisting of many platelets are imaged. Importantly, sharp edges can be observed, and cross-section analysis reveals overall thickness profiles of the domains (Figure S2). The heights measured vary slightly, from 3 to 5 nm, which are thinner than the ML estimated from the main absorption band above, suggesting that thinner platelets were sampled. We note that some products dispersions showed significant aggregation, and after drop casting, domains revealed stacks or clusters of platelets that revealed larger heights. To understand this, scanning electron microscopy (SEM) was used to image those dropcast substrates. Figure S3 shows the platelets and the 2D morphology, as well as grouped discrete aggregates, which were difficult to separate, and was attributed to either residue BE in the purified product, or the result of excess purification steps, as described below.

**Figure 3:**
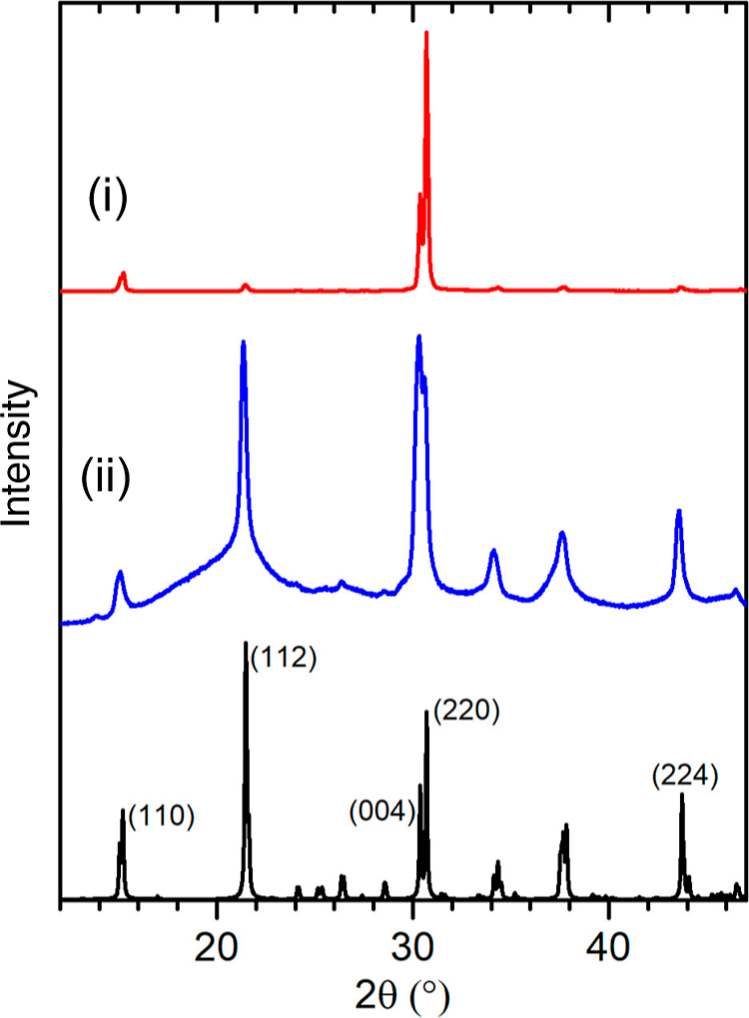
Representative powder XRD of BE-CsPbBr_3_ (i) and ODE-CsPbBr_3_ products (ii), as compared to an orthorhombic CsPbBr_3_ standard (97851-ICSD).

The crystalline nature of the CsPbBr_3_ products were studied by powder XRD. Figure 3 shows the XRD analysis of the BE (i) and ODE-CsPbBr_3_CsPbBr_3_ (ii), as compared to an orthorhombic CsPbBr_3_ bulk standard. Both products index with the orthorhombic standard with minimal variation to Bragg angles, but intensity ratios differed, as did the extent of Scherrer broadening. For example, the BE-CsPbBr_3_ (i) showed pronounced preferred orientation of the planes diffracting at 2θ = 30.4 and 30.7°, which correspond to (004) and (220) of the crystal. Clearly, the intensities do not match the standard and suggest not only that each platelet grows in the same orientation, but that each platelet is highly crystalline. It is important to note that preferred orientation in XRD can be a result of crystal growth, as well as an artifact produced by the way a sample self-assembles during drying, as well as by substrate type and sample-to-substrate interactions.^56^ We suspect that each of these factors influence the XRD shown here. The samples were prepared *via* drop-casting from a concentrated solution, and we assume they form into the irregular clusters shown by SEM (Figure S3). Nonetheless, a number of control experiments were performed to better understand the peak intensities and the origin of the preferred orientation. To test whether the intensity ratios could be an artifact of platelet drying on the XRD substrate, samples that were both drop cast from solution, and from dried powders, each of which showed similar intensities. In another control, a concentrated carbon black slurry with colloidal carbon (∼20 nm) was added to a toluene solution of purified BE-CsPbBr_3_, sonicated, and dropcast, with the aim of using the carbon to inhibit platelet stacking during drying. This however resulted in similar XRD signatures (Figure S4), suggesting that crystal orientation plays at least some role, and that growth occurs in the (004) and (220) directions of the platelets, of which (220) can be indexed to the longer dimension of the platelet. The thickness of the platelets, and thus, the planes of atoms in that direction are outweighed in terms of number and have lower Bragg intensities. The ODE-CsPbBr_3_, on the other hand, had intensity ratios consistent with that of bulk, as well as broadening consistent with the smaller and more isotropic dimensions.

The halide concentration of the platelets could also be fine-tuned, either by introducing iodide (I) into the synthetic solutions, or *via* HE, resulting in BE-CsPbBr_3–*x*_I_*x*_. Here, we focused on only the BE solvent since there are numerous examples of halide control in ODE-based products.^57^Figure S5 shows the PL for BE-CsPbBr_3–*x*_I_*x*_, synthesized by varying the [Br^–^]:[I^–^] feed ratio, with the corresponding XRD shown in Figure S6. The XRD signatures were consistent with those platelet morphologies shown above, and increased I-content (*x*) is indicated by shifts in 2θ. Products formed at low *x* showed the platelet-like preferred orientation; however, this was lost at high *x*. Alternatively, HE could be used to transform the BE-CsPbBr_3_*via* addition of I^–^ rich precursors^57^ or small organohalide molecules.^58,59^ Using a protocol recently developed in our laboratory,^51^ we found that the BE-CsPbBr_3_ platelets could undergo HE without disrupting the crystal structure and allowed for a broader control of composition than direct synthesis (Figure S7). A more detailed study of the synthesis of mixed halides and of HE in BE-CsPbBr_3_ are beyond the scope of this paper and will be reported elsewhere.

**Figure 4:**
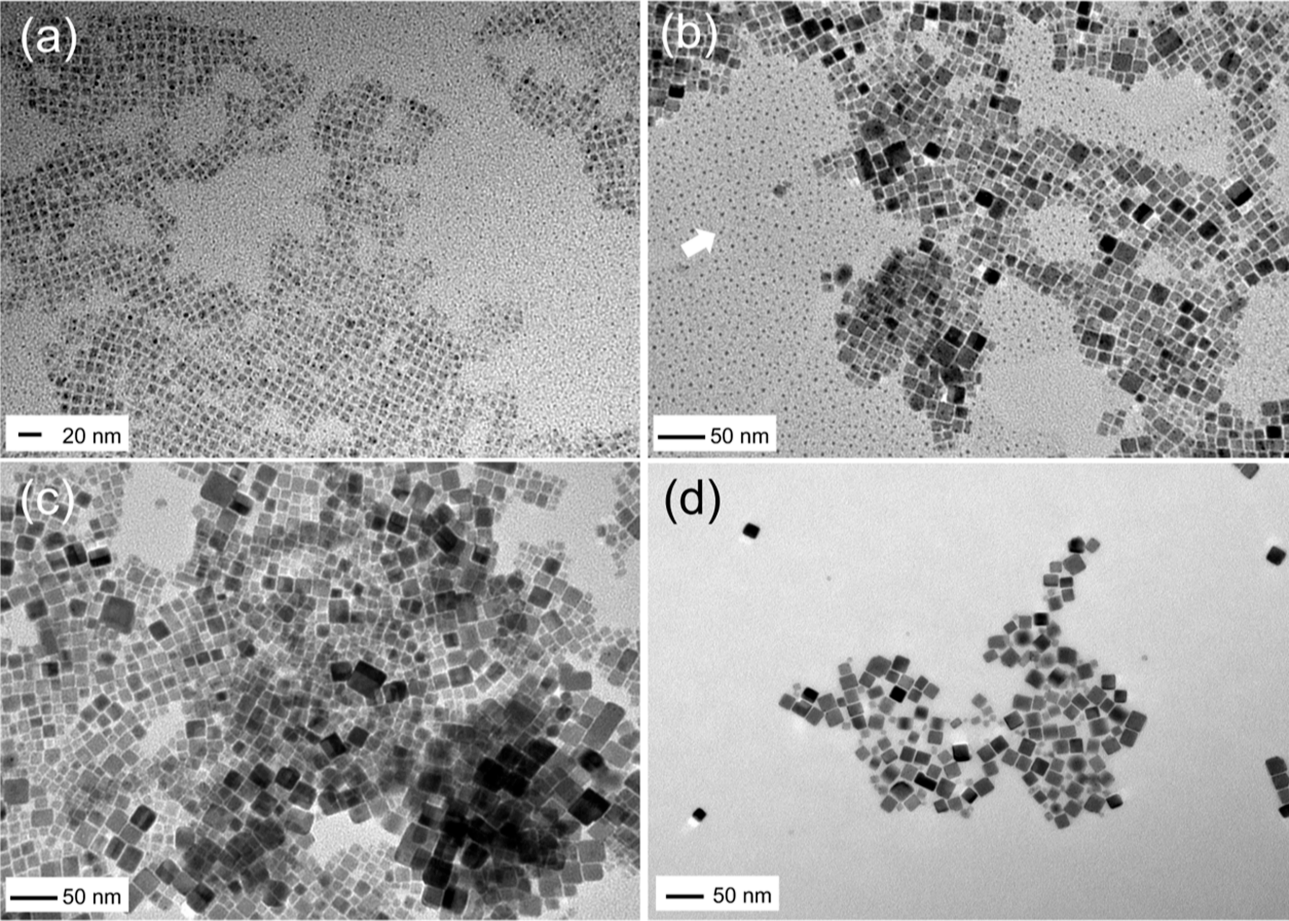
(a–d) Representative TEM micrographs of BE-CsPbBr_3_ products *via* hot-injection in BE. Samples collected from soluble (a, *l* = 6.8 ± 1.1 nm) and non-soluble fractions (b,c, *l* = 11.1 ± 3.5 nm) of the synthesis solution after centrifugation. Early reaction times show a high concentration of small clusters (arrow, *d* ∼ 3.5 nm) and one non-soluble product after 30 min annealing (d, *l*1 = 17.4 ± 2.6 nm, *l*2 = 10.4 ± 1.6 nm).

The novel component of this study is understanding the role of BE in the formation of CsPbBr_3_, and we next prepared BE-CsPbBr_3_ not with MWI heating but instead *via* hot-injection and convection. In contrast to MWI-based heating, in which all precursors are in “one-pot” and growth is facilitated or activated by heating, “hot-injection” introduces the final precursor at an elevated temperature, inducing burst nucleation and growth of what is typically a smaller and more monodispersed product. Figure 4 shows a set of TEM micrographs for BE-CsPbBr_3_ products collected after hot-injection and ∼1 min annealing at 160 °C (a–c), and after annealing for 30 min (d). One observation made after synthesis was that the product had both soluble and insoluble fractions after 1 min (Figure S8a). A TEM of the soluble portion is shown in Figure 4a, with small square crystals with edge lengths of *l* = 6.8 ± 1.1 nm visible. The insoluble portion (5 b,c) shows larger square platelets with *l* = 11.1 ± 3.5 nm. Both fractions showed smaller clusters or nuclei with diameters of *d* ∼ 3.5 nm and very uniform inter-cluster distances, see arrow. Based on the optical signature, which showed a band edge absorption at 500 (a–c) and 510 nm (d), these had thicknesses of ∼12 ML, respectively (Figure S8), making them much thinner than the MWI-based products.

**Figure 5:**
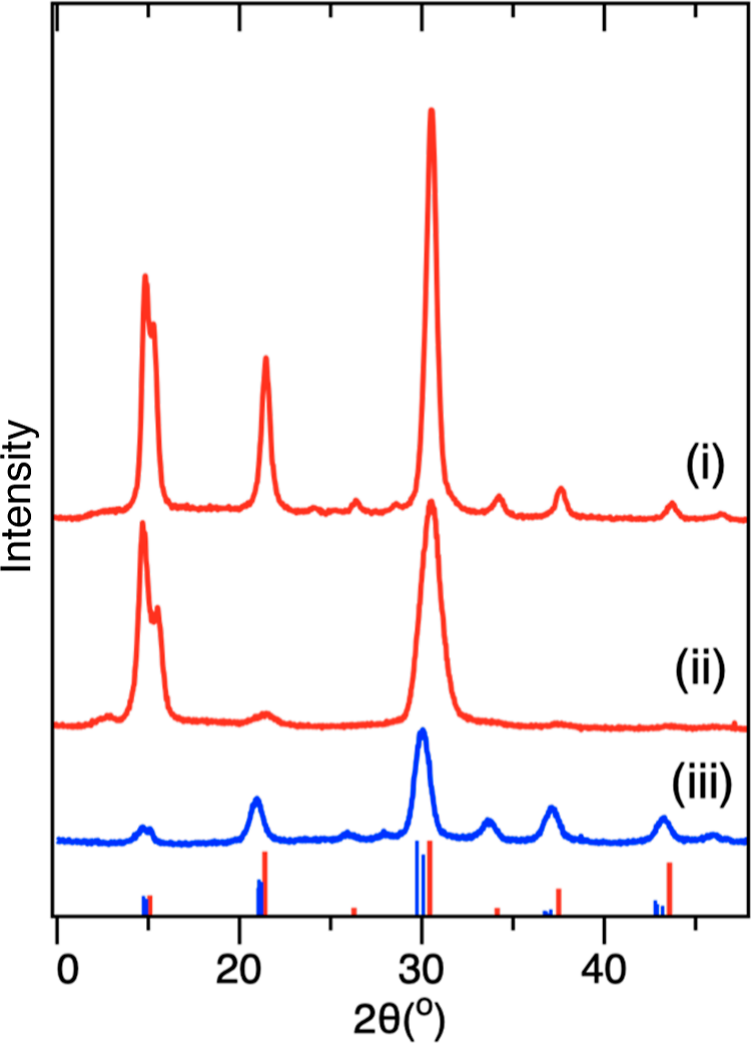
Representative powder XRD of BE-CsPbBr_3_ hot-injection products for soluble (i) and insoluble (ii) fractions and ODE-CsPbBr_3_ (iii) after ∼1 min annealing. Reference patterns for cubic (1533063-COD, red) and orthorhombic CsPbBr_3_ (97851-ICSD, blue).

Figure 5 shows the powder XRD for BE-CsPbBr_3_ soluble (i) and insoluble (ii) products from hot-injection, as compared to ODE-CsPbBr_3_ (iii). Compared to the MWI BE-CsPbBr_3_ products (Figure 4), these showed more cubic crystal characteristics, with the insoluble products (ii) showing some preferred orientation. The ODE-CsPbBr_3_ similarly showed cubic similarities but with slight 2θ shifts that may suggest some orthorhombic features.

**Figure 6:**
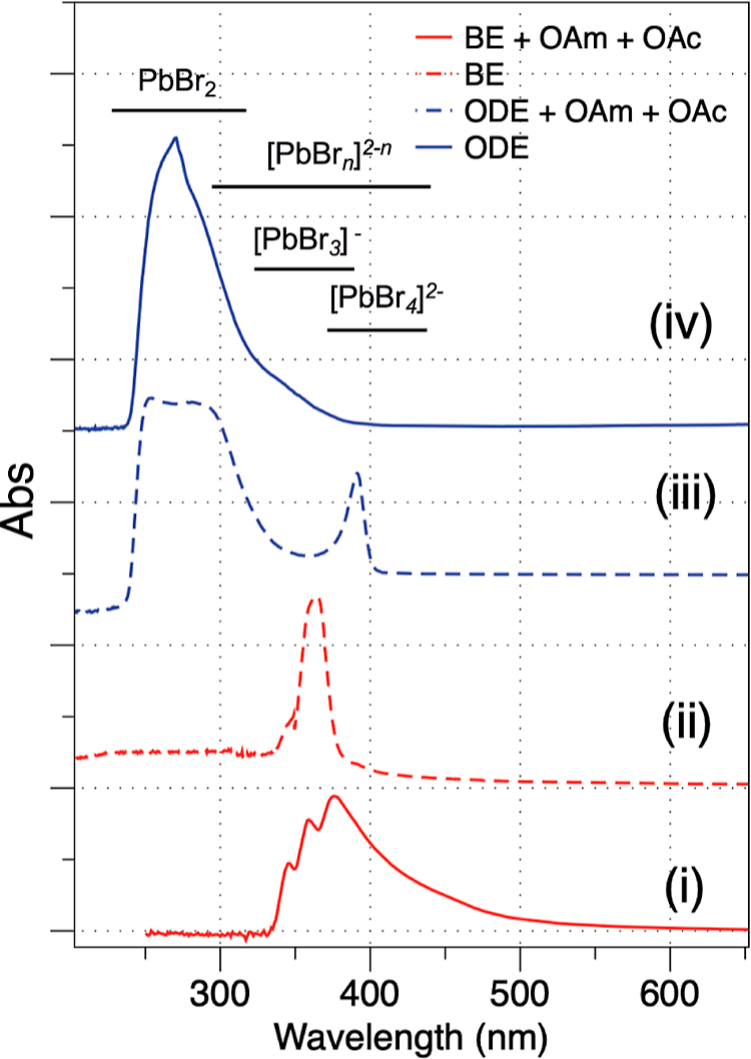
Normalized UV–vis of PbBr_*n*_^2–*n*^ formed *via* PbBr_2_ dissolved in BE (i), BE + OAc + OAm (ii), ODE + OAc + Oam (iii), and ODE (iv) at 140 °C under vacuum for 1 h and then cooled to room temperature. Approximate absorption regions for different PbBr_*n*_^2–*n*^ complexes are shown. Spectral ranges used vary due to solvent absorption.

We hypothesize that the reason why BE and ODE produce different morphologies is due to the different fractions of precursor types at the time of nucleation, as well the affinity of the solvent to Pb^2+^. Figure 6 shows the UV–vis of PbBr_2_ solid dissolved in BE (i) and ODE (iv), and mixtures containing ligands, BE + OAm + OAc (ii), as well as ODE + OAm + OAc (iii). The absorptions observed are categorized broadly as exfoliated PbBr_2_ solids, which may be 2D in nature,^53^ and multiple PbBr_*n*_^2–*n*^ lead plumbate complexes which are often defined as PbBr_6_^4–^, PbBr_3_^–^, and PbBr_4_^2-^.^52,54^ Comparing (i) to (iv) suggests that BE is more effective at dissolving PbBr_2_ to PbBr_*n*_^2–*n*^ than ODE. This was also physically observed in the experiment, where BE dissolved more PbBr_2_ salt. Second, the addition of OAm and OAc further dissolves the solids in the case of ODE and shifts the absorption wavelengths in BE. Both solvent plus ligand mixtures show high concentrations of PbBr_*n*_^2–*n*^, likely the result of ligand coordination to Pb^2+^ and substitution of one or more bromides (see below), with ODE + OAm + OAc still showing a considerable percentage of insoluble PbBr_2_. Whether or not this solid PbBr_2_ is incorporated into forming the nano CsPbBr_3_ is likely dependent on the effect of the synthetic temperature on dissolution equilibrium.

This insight suggests that BE coordinates and solvates PbBr_2_ better than ODE, which is understandable considering its π-rich nature. Researchers have studied the solvent effect of perovskite formation previously, especially as it relates to thin-film formations of methyl ammonium halides (MAPbX_3_) using polar solvents,^43^ as well as the role polar solvents can play in assembling or transforming smaller platelets into larger ones.^21^ We note that BE has been used in nanoparticle synthesis before, especially in the recent synthesis of Qdot heterostructured libraries.^60−62^ By using a competitive assay between solvents and halides, Benesi-Hildebrand analysis can be used to approximate coordination strength by way of estimating equilibrium constant (K) and Guttmann donor number (DN), which measures PbBr_*n*_^2–*n*^ concentration and type in the presence of excess halides by way of UV–vis.^43,44,52,63^ For instance, Loo and co-workers compared solvent dielectric constants and DN with either crystal growth or thin-film growth mechanisms and showed that while ε values did not predict growth, DN > 15 consistently resulted in thin-film growth, whereas higher numbers consistently showed crystal growth.^43^ Thus, higher DN solvents, typical polar and strong Lewis bases, coordinate favorably with Pb^2+^, resulting in PbBr_*n*_^2–*n*^ with higher *n* and more crystal growth (ideal building blocks), whereas DN < 15, coordinate weakly with Pb^2+^, resulting in lower *n* and more amorphous or thin-film growth.^43^ Till date, most of the analysis is compared to polar solvents, like DMF and DMSO, both of which dissolve PbBr_2_ and determine plumbate equilibrium.^43,44,52^ Both BE and ODE are considerably less polar that many of these solvents used for perovskite growth. Figure S9 shows Benesi-Hildebrand assay used to compare BE and ODE, to DMF, where PbI_2_ and MAI were used in place of PbBr_2_ and MABr. Both assays resulted in apparent formation constants (*K*) comparable to other non-coordinating solvents, where *K*_BE_ ∼ 74 M^–1^ and *K*_ODE_ ∼ 84 M^–1^, suggesting that BE prohibits PbBr_*n*_^2–*n*^ formation more than ODE, with both much weaker than DMSO (*K*_DMSO_ ∼10, Figure S9). The composition of the final organic capping layer of BE-CsPbBr_3_ was also studied *via* FTIR and shown in Figure S10. Vibrations attributed to BE adsorbed to the CsPbBr_3_ interface were consistently observed, further suggesting coordination.

While the DN and *K* values aid in understanding the PbBr_2_ dissolution, the cesium oleate precursor is also important and can have different temperature-dependent and stoichiometry-related solubility. Control experiments fully solubilizing cesium oleate (Cs^+^-OAc) at room temperature were performed, which used high OAc-to-Cs molar ratios, following a method recently described.^48^ The products of that control synthesis using MWI had more soluble final products, but the platelet morphology and XRD intensity ratios persisted (Figure S11). This Cs precursor was also used in the hot-injection synthesis described above.

**Figure 7:**
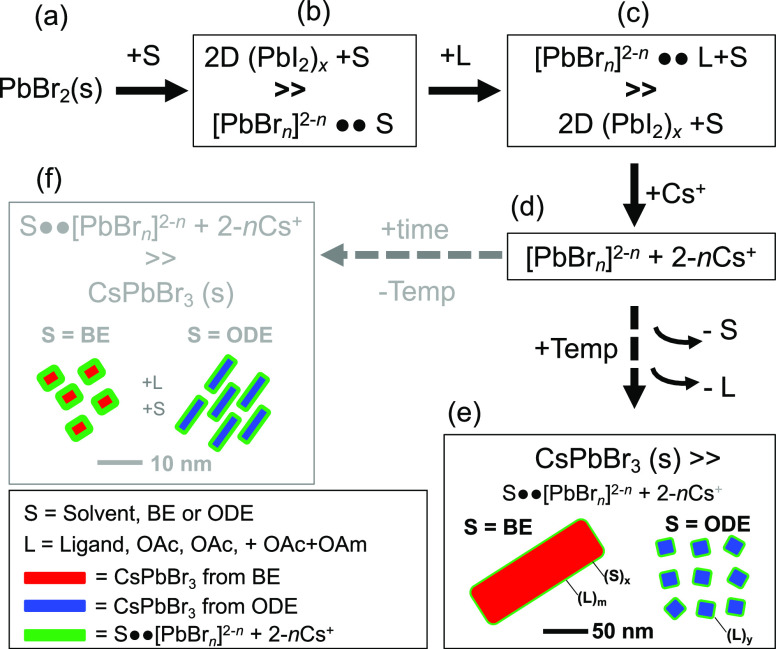
Idealized mechanism schematic for the growth of CsPbBr_3_ using BE or ODE solvents, showing the precursor (a), intermediate complexes (b–d), and products (e,f).

Considering the procedural steps employed in this study, and the findings above, Figure 7 idealizes the mechanism for CsPbBr_3_ growth. The dissolution of PbBr_2_ salt (a) in a solvent (S = BE or ODE) produces two intermediates, 2D, exfoliated (PbBr_2_)_*x*_ solid layers solvated by S, as described recently,^53^ and the PbBr_*n*_^2–*n*^ plumbates of various coordinations, such as PbBr_4_^2–^, PbBr_3_^–^, and so forth.^52,54^ Here, the PbBr_*n*_^2–*n*^ may have a Br^–^ substituted by S, which is not charged (b). Upon addition of ligands (L = OAm, OAc, and OAm + OAc), the equilibrium shifts to forming a higher percentage of PbBr_*n*_^2–*n*^ afforded by strong L-to-Pb^2+^ coordination (c), which breaks the PbBr_2_ into smaller fragments or lower molecular weight polyhedra. Upon the addition of Cs^+^ (d), the PbBr_*n*_^2–*n*^ polyhedra are electrostatically attracted to one another, forming 2–*n*Cs^+^PbBr_*n*_^2–*n*^ complexes, but still under the coordination of excess L and S. In this study, steps a–c (precursor preparation) occur over the course of an hour, whereas step (d) occurs over a few minutes before heating in the case of MWI heating or within seconds during hot-injection. Upon heating, the 2–*n*Cs^+^PbBr_*n*_^2–*n*^ complexes loose coordinating S as well as L and are consumed producing CsPbBr_3_ perovskite platelets (e). Loss of coordinating solvent during heating is often observed in the formation of perovskite thin films from polar solvents; however in this study, loss of solvent refers to those molecules that were either coordinating to the crystal or separating intermediate plumbates. It is possible that BE DN numbers likely reside in the thin-film growth regime, as described above, resulting in the fact that large mesoscale platelets are observed with prolonged MWI heating and smaller square platelets are formed *via* quick hot-injection, while ODE produces smaller crystallites of similar sizes for both heating conditions. Also of importance is the temperature used as it will influence the equilibrium between PbBr_*n*_^2–*n*^ types and CsPbBr_3_ crystallization in the presence of S and L, promoting PbBr_*n*_^2–*n*^ at lower temperatures.

Interestingly, if the reaction is held at step (d) for long periods of time (days) at room temperature (f), then differences between BE and ODE can also be observed. Kinetically, the BE-CsPbBr_3_ formed slower and resulted in thinner CsPbBr_3_ (Figure S12), where smaller crystallites are formed that have well-defined inter-crystal distances, which we attribute to repulsion from coatings of charged PbBr_*n*_^2–*n*^ at the interface. A TEM image of these is shown in Figure S13. The slower kinetics and smaller crystal size (*d* ∼ 3 nm) in the case of BE at room temperature again suggest strong coordination to PbBr_*n*_^2–*n*^, the release of which is more sensitive to temperature. ODE on the other hand formed uniform rodlike structures with lengths < 15 nm. This final point suggests that judicious selection of both solvent and modest temperature changes^64^ may allow for a wealth of morphologies to be formed and controlled, which is part of our ongoing work and will be reported elsewhere.

## Conclusions

Taken together, a synthesis route for CsPbBr_3_ nanoplatelets has been described, in which choice of non-polar solvents and heating methods can be used to control the morphology. The findings demonstrate that combining BE, a one-pot mechanism, and MWI heating prove effective at influencing nucleation and growth to the point of forming highly crystalline platelets, with lateral dimensions of 20–500 nm, and relatively thick, ∼15 ML, thicknesses. These platelets show preferred orientation in XRD signatures along the (220) and (004) planes. Synthesis *via* hot-injection with BE also leads to platelets but in a more uniform square shapes, ∼17 nm lengths and ∼2 ML thicknesses. On the contrary, use of ODE results in small crystallites, ∼10 nm, in both heating approaches. The ability of the solvent and solvent plus ligand mixtures to dissolve PbBr_2_ salt into varied PbBr_*n*_^2–*n*^ plumbates was studied and showed that BE is more effective, due in large part to its π-donating character and coordination to Pb^2+^. The compositions of the BE-CsPbBr_3_ could be tailored by adding iodine either *via* synthesis upon addition of PbI_*n*_^2–*n*^ during synthesis or *via* HE.

## Supporting Information Available

Additional TEM, SEM, AFM, XRD, and FTIR results and characterization of BE-CsPbBr_3–*x*_I_*x*_ products (PDF)
