# Supplementary material for: Tailoring CsPbBr3 Growth via Non-Polar Solvent Choice and Heating Methods
Source: Langmuir. 2022 Jul 21;38(30):9363–71. doi: 10.1021/acs.langmuir.2c01214 (PMC9352358; doi:10.1021/acs.langmuir.2c01214)
Supplement: Supplementary file 1 — la2c01214_si_001.pdf [file la2c01214_si_001.pdf]

Tailoring $CsPbBr_3$ Growth Via Non-Polar Solvent Choice and Heating Method

Hediyeh Zamani, Tsung-Hsing Chiang, Kaylie R. Klotz, Annie J. Hsu, Mathew M. Maye*
Department of Chemistry, 111 College Place, Syracuse University, Syracuse, New York, U.S.A.
**mmmaye@syr.edu*

Supporting Information

Supporting Figures

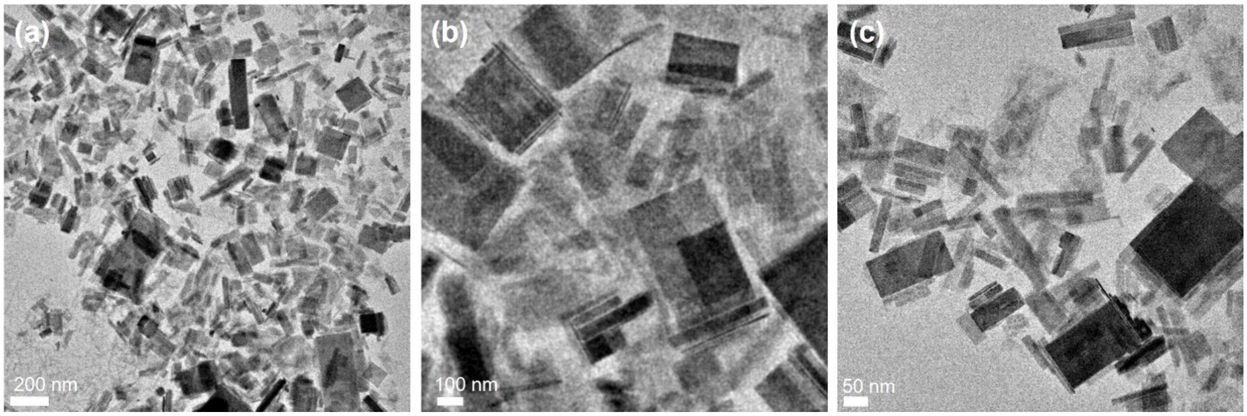

Figure S1: Additional TEM micrographs of BE-$CsPbBr_3$ nanoplatelets prepared in BE solvent and under microwave irradiation at 160 °C.

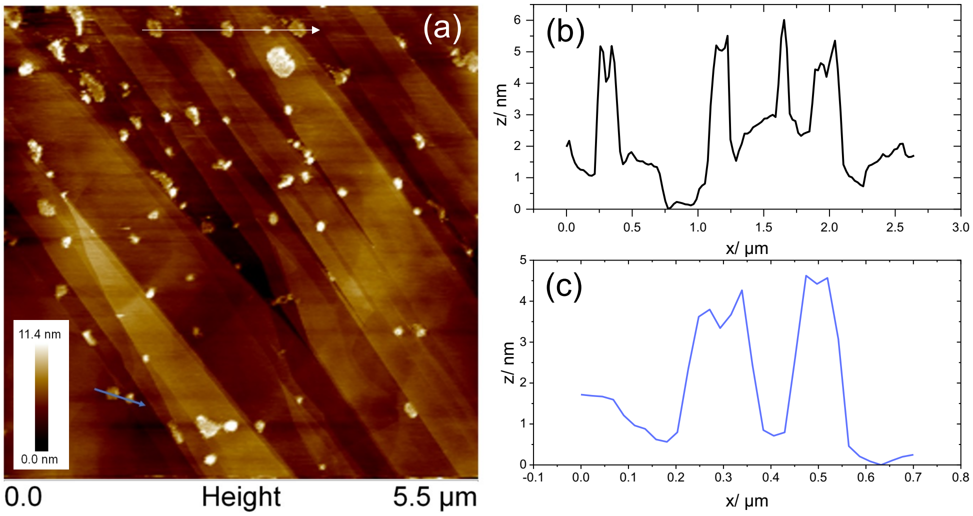

Figure S2: Representative tapping mode AFM image (a) and corresponding cross-section analysis (b-c) of two regions (arrows) of BE-$CsPbBr_3$ nanoplatelet domains drop cast onto HOPG.

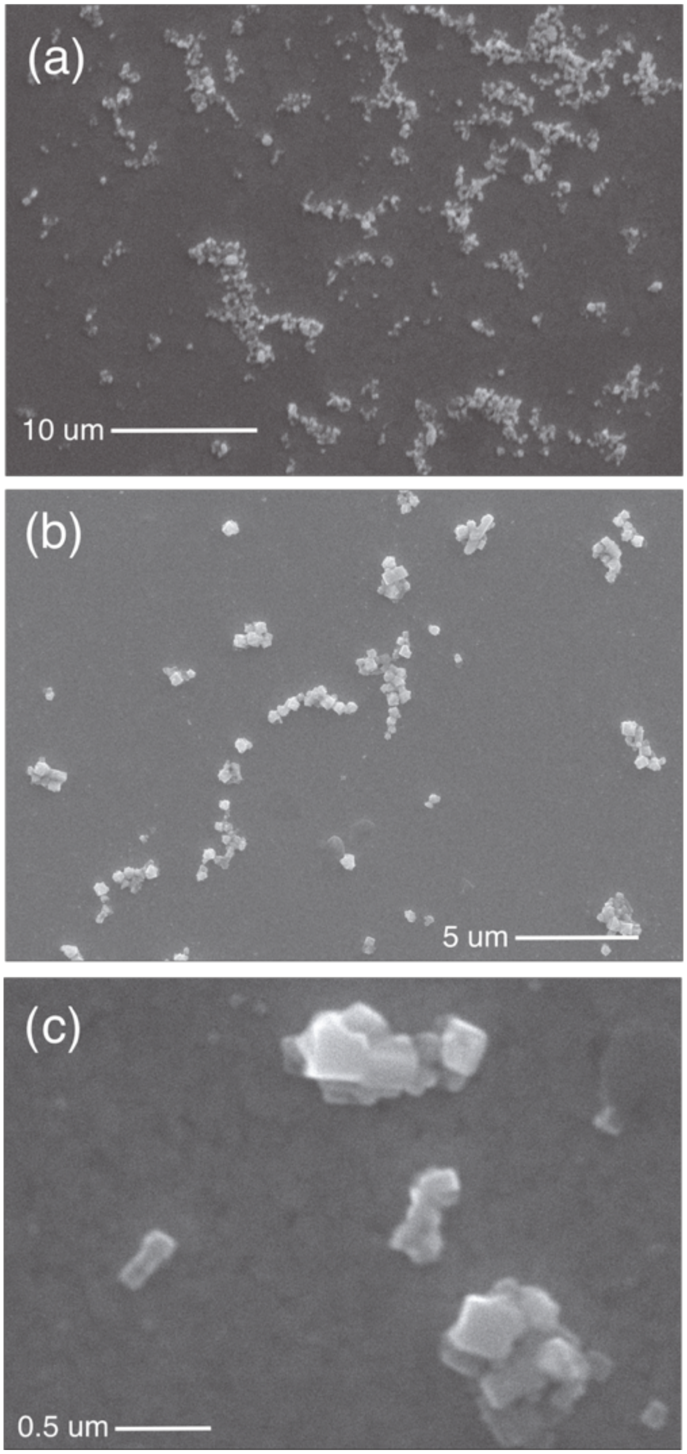

Figure S3: Representative SEM micrographs of purified BE-$CsPbBr_3$ dropcast onto HOPG and imaged at increasing magnifications (a-c).

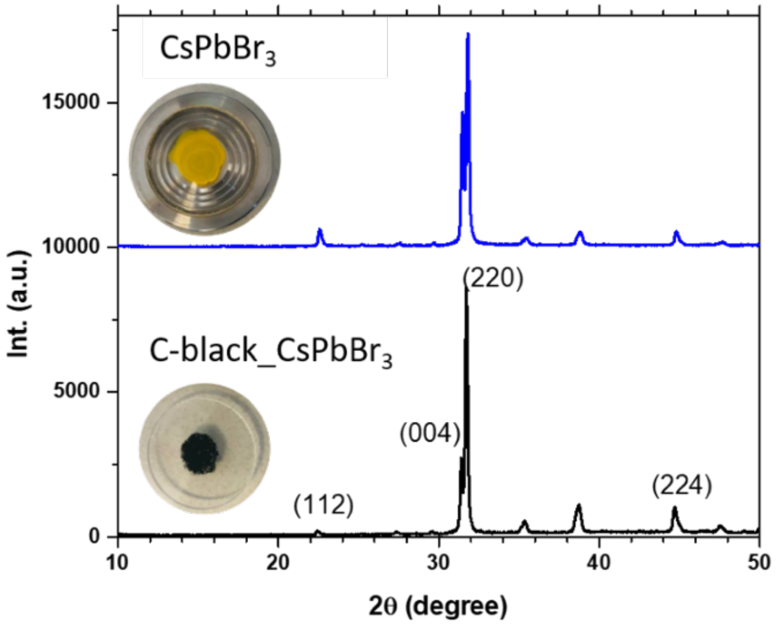

Figure S4: Control experiment XRD results comparing the purified BE-$CsPbBr_3$ nanoplatelets prepared (top) and same nanoplatelets with 50 µL of 3 g/L carbon black added and sonicated before drop casting (bottom), testing whether or not the nanoplatelets stacked on the XRD holder, which was found to not be the case, due the similarity of the two results. Orthorhombic reference planes shown for illustration only.

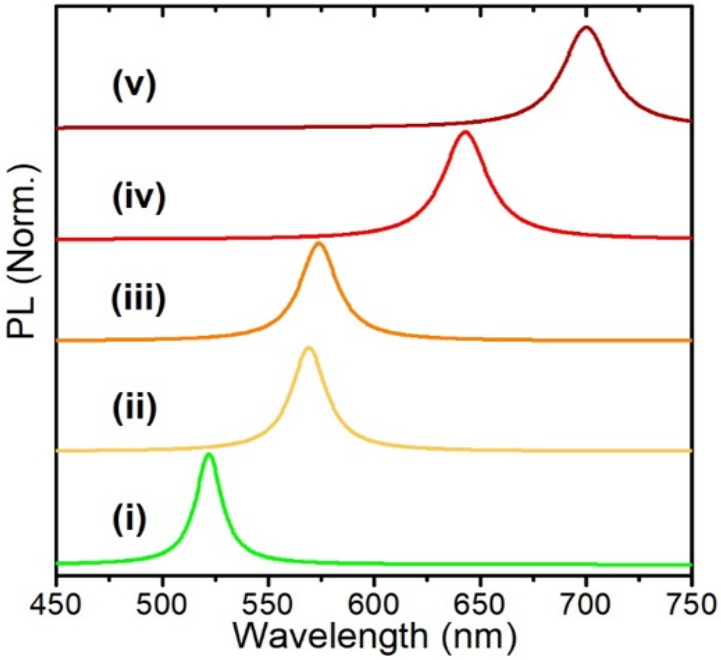

Figure S5: PL emission results of BE-$CsPbBr_{3-x}I_x$ formed via the one-pot MWI heating (160 °C) synthesis using increasing concentrations of PbI_n^{2-n} (i-v) complexed with BE and OAm.

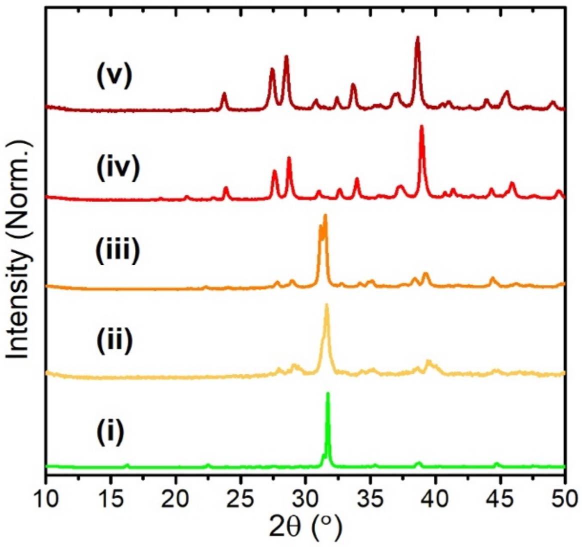

Figure S6: XRD results of BE-$CsPbBr_{3-x}I_x$ formed via the one-pot MWI heating (160 °C) synthesis using increasing concentrations of PbI_n^{2-n} (i-v) complexed with BE and OAm.

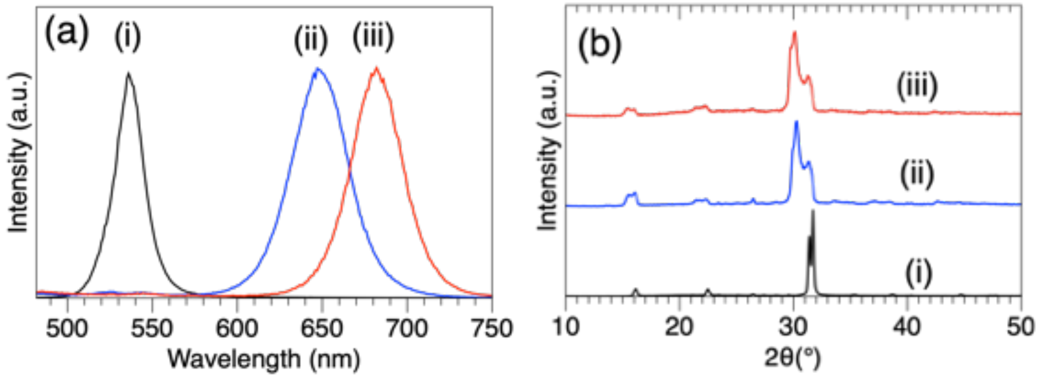

Figure S7: PL emission (a) and XRD (b) of BE-$CsPbBr_3$ nanoplatelets before (i), and after halide exchange with two consecutive washing steps with OAm-PbI_2 (ii-iii) following our recent work.[1]

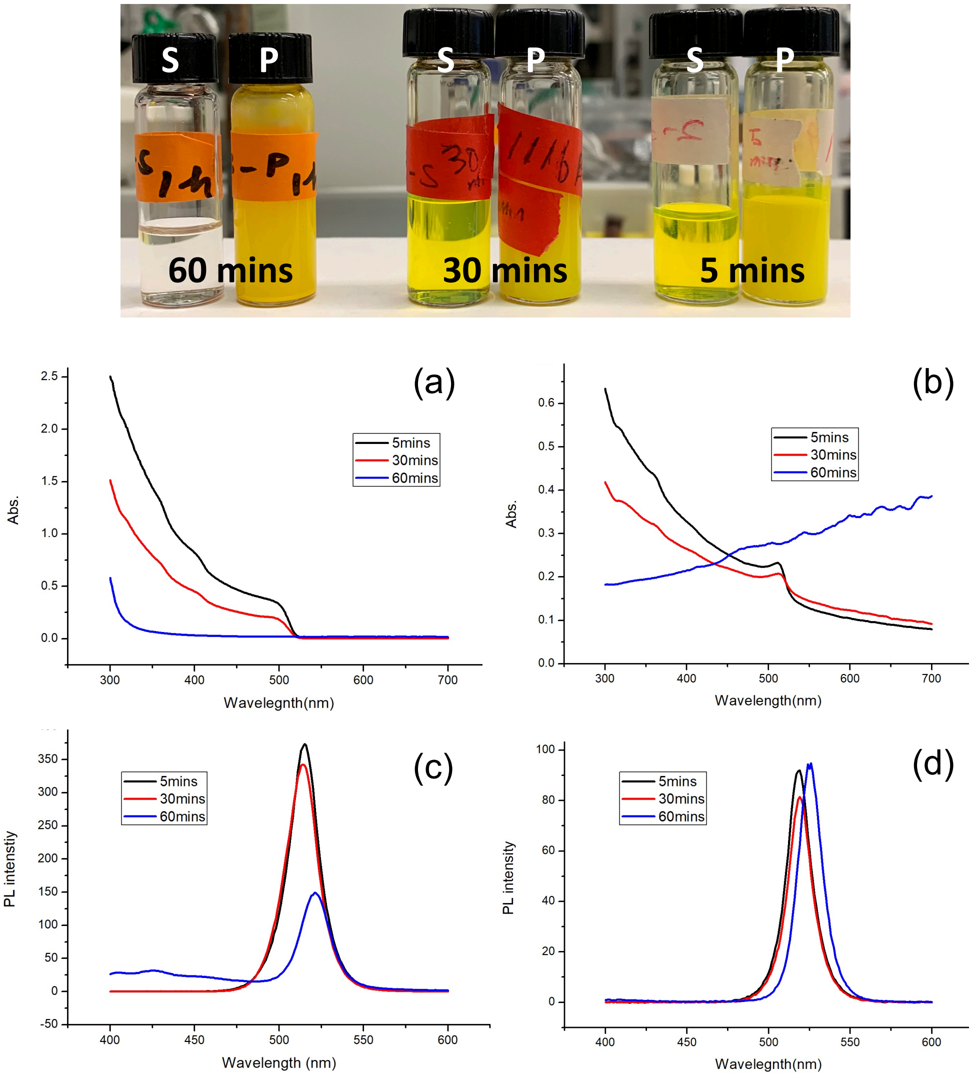

Figure S8: (Top Panel) Photograph of supernatant (S) and precipitate (P) BE-$CsPbBr_3$ products from hot-injection synthesis after 60, 30, and 5 min annealing times. (Bottom Panel). UV-vis of BE-$CsPbBr_3$ products supernatant (a) and precipitate (b) after 5, 30 and 60 min annealing times, with corresponding PL results for supernatant (c) and precipitate (d).

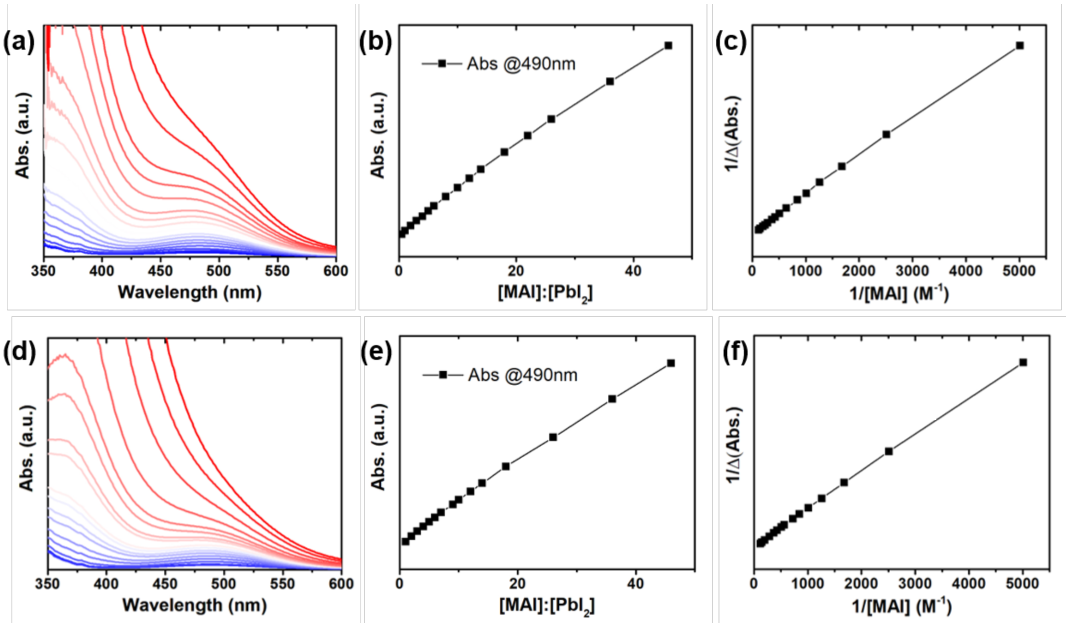

Figure S9. Benesi-Hildebrand analysis of PbI_n^{2-n} complex formation by reacting PbI_2 (dissolved in BE) with increasing [MAI] (diluted in BE) (a-c), compared to a 50:50 (v/v) BE:ODE mixture (d-f). In analysis in plot (c) and (f) results in approximate equilibrium constant (K) of ~74 and 86 M^{-1}, respectively, indicating that BE hinders plumbate formation more than ODE. We note that PbI2 and MAI were used in place of $PbBr_2$ and MABr due to availability of chemicals, and that those equilibrium may be different. Typical K for strongly coordinating solvents, like DMSO are ~10, and non-coordinating, like PC (propylene carbonate), are ~95. [2]

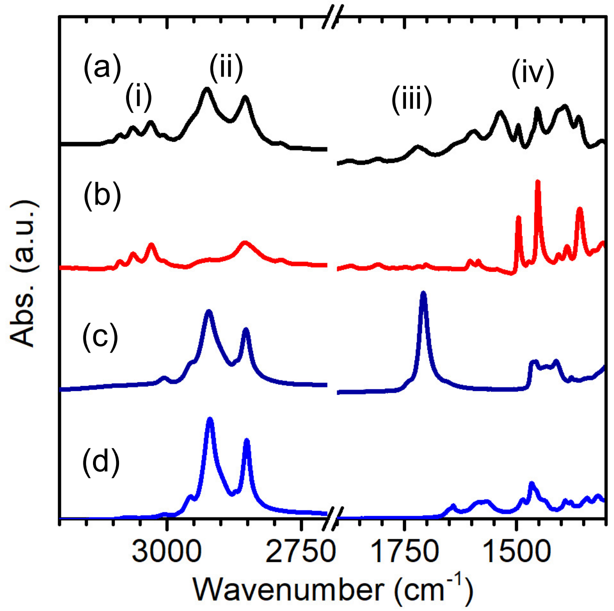

Figure S10: FTIR of purified BE-$CsPbBr_3$ product (a), BE, (b), OAc (c), and OAm (d), and highlighted regions of interest, C-H benzyl rings (i), aliphatic C-H (ii), C=0 (iii), and C=C (iv).

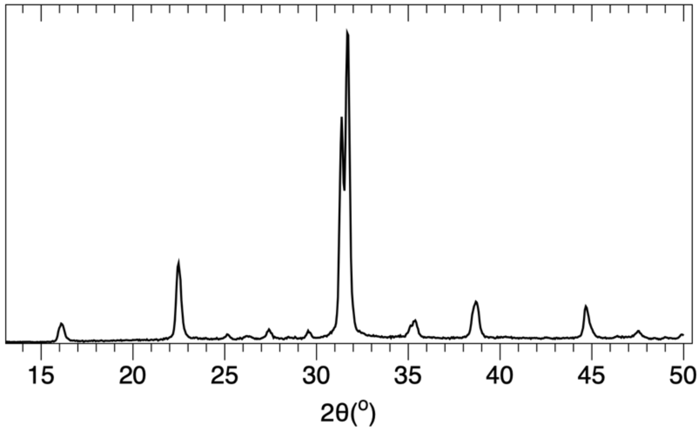

Figure S11: XRD results of the BE-$CsPbBr_3$ nanoplatelet products produced at [OAc]:[Cs^+] = 5, demonstrating that platelet formation was still observed with more soluble precursors.

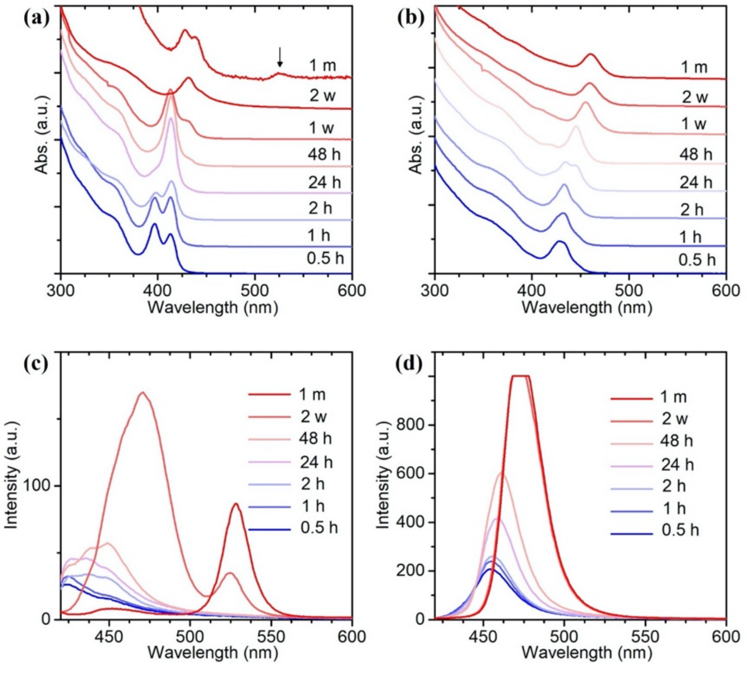

Figure S12: UV-vis and PL monitoring of BE- (a,c) and ODE-$CsPbBr_3$ growth over time at room temperature.

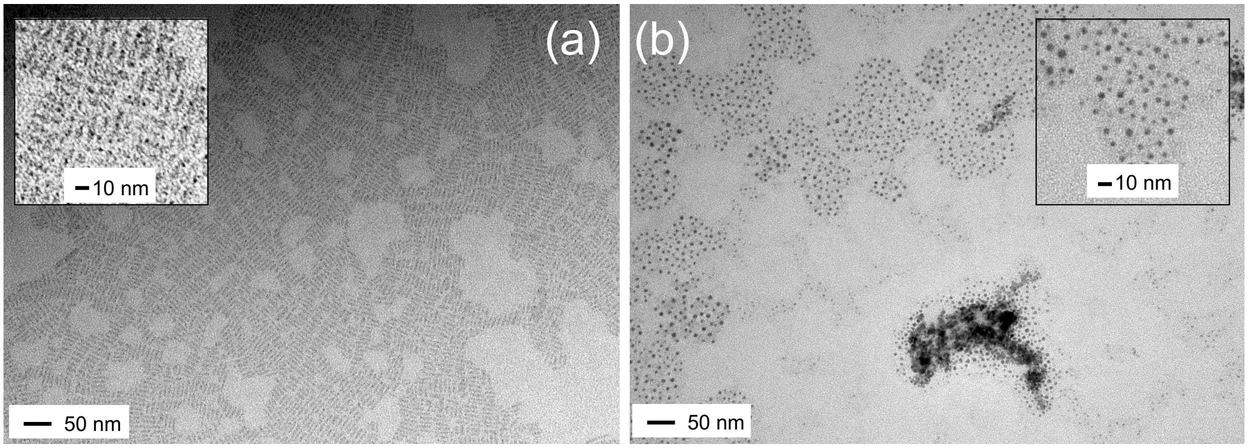

Figure S13: Representative TEM micrographs of ODE- (a) and BE-$CsPbBr_3$ (b) products formed at room temperature after 24 h reaction. Inserts: Enlarged areas within micrographs.

<u>**Supporting References:**</u>

(S1) Ripka, E. G.; Deschene, C. R.; Franck, J. M.; Bae, I.-T.; Maye, M. M. Understanding the Surface Properties of Halide Exchanged Cesium Lead Halide Nanoparticles. *Langmuir* **2018**, *34* (37), 11139–11146. https://doi.org/10.1021/acs.langmuir.8b02148.

(S2) Hamill, J. C.; Schwartz, J.; Loo, Y.-L. Influence of Solvent Coordination on Hybrid Organic–Inorganic Perovskite Formation. *ACS Energy Letters* **2018**, 92–97.
